# Optimizing the usage of pupillary based indicators for cognitive workload

**DOI:** 10.16910/jemr.14.2.4

**Published:** 2021-06-11

**Authors:** Benedict C. O. F. Fehringer

**Affiliations:** University of Mannheim, Germany

**Keywords:** Eye tracking, pupillometry, Index of Cognitive Activity (ICA), Index of Pupillary Activity (IPA), cognitive workload, R-Cube-Vis Test

## Abstract

The Index of Cognitive Activity (ICA) and its open-source alternative, the Index of Pupillary Activity (IPA), are pupillary-based indicators for cognitive workload and are independent of light changes. Both indicators were investigated regarding influences of cognitive demand, fatigue and inter-individual differences. In addition, the variability of pupil changes between both eyes (difference values) were compared with the usually calculated pupillary changes averaged over both eyes (mean values). Fifty-five participants performed a spatial thinking test, the R-Cube-Vis Test, with six distinct difficulty levels and a simple fixation task before and after the R-Cube-Vis Test. The distributions of the ICA and IPA were comparable. The ICA/IPA values were lower during the simple fixation tasks than during the cognitively demanding R-Cube-Vis Test. A fatigue effect was found only for the mean ICA values. The effects of both indicators were larger between difficulty levels of the test when inter-individual differences were controlled using z-standardization. The difference values seemed to control for fatigue and appeared to differentiate better between more demanding cognitive tasks than the mean values. The derived recommendations for the ICA/IPA values are beneficial to gain more insights in individual performance and behavior during, e.g., training and testing scenarios.

## Introduction

Pupillary responses are related to processing load, cognitive demands of memory, language processing, reasoning, and perception (see Beatty, 1982; 17). Although Just and Carpenter (15) pointed out that pupillary responses are only a correlate of cognitive demands but not causally related, Kahneman stated that the “dilation of the pupil is the best single index [for effort]“ (17, p. 18). In general, greater amounts of pupil dilations are expected to indicate more difficult tasks (see 1). For example, Karatekin et al. ( 18) showed an increasing pupil dilation for more difficult tasks in a digit span-working memory test.

However, studies concerning potentially influencing factors such as fatigue or inter-individual differences in the characteristics of pupil diameter changes yielding ambiguous results. The present study investigated how these factors influence the pupil diameter and its changes and whether inter-individual differences can be controlled. Light as an additional influencing factor (36) was taken into account in the present study by using the Index of Cognitive Activity (22) as a pupillary based indicator of cognitive workload that controls for light changes as well as the Index of Pupillary Activity (IPA, 6), which is an open-source alternative of the ICA. Pupillary based measures are appropriate indicators for cognitive workload. For example Krejtz et al. ( 19) showed their effectiveness in comparison with microsaccades.

### The Index of Cognitive Activity and the Index of Pupillary Activity

The Index of Cognitive Activity (ICA) was invented by Marshall and can be computed by a patented method evaluating pupil dilations ( 21). This indicator is robust with respect to light changes and increases with task difficulty (22). Further studies supported the ICA as an appropriate measure for cognitive effort (e.g. 3; 26). In the study of Demberg (3), participants performed a simulated driving task as well as a language processing task. She found evidence that the ICA was more appropriate than conventional pupil dilation measures in their response to cognitive processing. The ICA also indicated strategy switches that lead to lower cognitive demands during task performance. Marshall (22) could find a self-reported change of how a participant conducted a series of tasks by a decreasing ICA value (see also 23). Furthermore, Schwalm et al. (35) showed that the ICA increased in a driver scenario where participants were performing lane change tasks in a dual task scenario. The higher the mental demand, the more the ICA increased.

The severe disadvantage of the ICA is that the underlying algorithm is unpublished and, therefore, not verifiable. The Index of Pupillary Activity (IPA, 6) is an open-source alternative that imitates the ICA and has a fully documented algorithm. Recently, Duchowski et al. (5) published an alternative version of the algorithms, which considers low and high frequencies of pupillary oscillation together (LHIPA). Since the goal of the present study was an evaluation of the (currently) more popular ICA, the original version of the more similar IPA was considered in the following analyses.

### Pupil diameter changes as indicator for fatigue

The pupil diameter is sensitive for fatigue. The more fatigue a person is, the smaller is the pupil diameter (see 1). This could be supported by Hopstaken et al. (13) who found a decreasing pupil diameter baseline with increasing testing time. However, they could also show an increasing pupil diameter baseline if task engagement increased, despite of fatigue. The results are similar if pupil diameter changes are analyzed (28). Clear evidence about the relation between the ICA (and IPA) and fatigue is missing. The results of a long-term study with three participants showed only inconsistent results, which were also hard to interpret due to missing performance information (21).

### Inter-individual comparisons of pupil diameter changes

Different ability levels are indicated by different pupil diameter changes with smaller pupillary responses for participants with higher intelligence or higher expertise than for participants with lower intelligence or expertise as a general pattern (e.g. 2, 32). However, this only holds true for tasks that are manageable for all participants. If tasks are too difficult for participants, pupil dilation can decrease (e.g. 12; 37). Zekveld and Kramer ( 40) varied the intelligibility range of masked speech and demonstrated in a sample of 37 participants that participants with lower ability reported more often to give up in the most difficult condition than participants with high ability. For the low ability group, pupil dilations were smaller for low intelligibility than for medium intelligibility, whereas high ability participants showed the opposite pattern. Therefore, pupillary responses are thought to indicate capacity utilization rather than absolute processing demands (16). For more demanding cognitive tasks, more resources can only be allocated if the participant has the required ability. In case of excessive demands, the mental effort can decline because of disengagement. However, it is not known yet whether the maximum pupil diameter changes are equal for all participants or whether they change with the amount of allocated resources. Therefore, comparisons of pupil diameter changes of participants with different abilities might be misleading, if the participant’s ability in relation to the task difficulty is not known. One solution could be that the pupil diameter values are standardized before such comparisons are conducted.

### Variability of pupil diameter changes between both eyes

The amount of variability between both eyes might also be an indicator of cognitive workload. In a general statement, Kahneman (17) pointed out that “the reduction of autonomic variability during task performance is apparently a general effect: rhythmic contractions and dilations of the pupil, which are prevalent at rest, are virtually abolished during the performance of mental arithmetic.” (p. 17). Especially the relation between reduction of heart rate variability and cognitive workload could be found in several studies (e.g. 4; 7; 24). Therefore, the pupil changes of both eyes might be also more “in phase”, if the cognitive demand increase. Hence, the absolute difference between the values of the pupil diameter changes of both eyes were investigated as an alternative measure in contrast to averaging over the values of the left and the right eye. The lower the absolute difference is, the higher the expected cognitive effort should be.

### Summary and hypotheses

Pupil diameter changes seem a promising indicator for cognitive workload but appear to be influenced by several factors, such as light changes, fatigue, and the interaction between task demands and individual differences in ability. The Index of Cognitive Activity (ICA) as well as the Index of Pupillary Activity (IPA) control for light influences and are valid indicators of cognitive effort. Fatigue is generally associated with decreasing pupil diameter changes but these may increase if the task engagement of the participant increases. The investigation of inter-individual differences as well as different demanding tasks can be hampered by the different amount of resources that a single participant is able to allocate for solving a certain task. In case that the maximum ICA/IPA value depends on the interaction between task engagement and ability differences, a standardization of the measured values might provide more valid results. Finally, the variability of both eyes regarding the pupil dilation might be reduced for more demanding tasks because of a reduction of the autonomic variability. Hence, the difference between the pupil diameter changes between the left and the right eye might be an additional indicator of cognitive workload.

The goal of the present study was to investigate how tasks differing in cognitive demand, fatigue, standardization (to control for inter-individual differences in ability) and the consideration of the difference between the eyes affect the ICA and the IPA values. It was expected that all pair comparisons between task groups that differ in their cognitive demand would show higher ICA/IPA values and lower variability between the eyes for the more demanding tasks. Pair comparisons between task groups to investigate the fatigue effects were unspecified due to the inconsistent results in previous studies.

## Methods

### Participants

The study was conducted with N = 55 participants, 43 female and 12 male. On average, the participants were 21 years (M = 21.07, SD = 3.84), with the youngest being 18 and the oldest 39. Further 14 participants were excluded due to invalid eye tracking data and because of erroneous recorded data. All participants were students at a German University. They provided informed consent and received course credit.

### Materials

The short version of the R-Cube-Vis Test (11) was administered as the performance test. The test measures the main factor of spatial thinking, visualization, and it was constructed especially for the usage of eye tracking and pupillometry. Other standard tests for spatial thinking are limited, if eye tracking is used due to heterogenous stimulus materials, overlapping of relevant areas, and too complex items. The R-Cube-Vis Test overcomes these restrictions and was, therefore, utilized in the present study. The short version consists of 60 items showing two Rubik’s cubes. The left cube is shown in a solved state whereas the right cube has one or two of its elements rotated. Participants had to decide whether both cubes are possibly the same except for the rotated elements (possible vs. impossible items).

**Figure 1.**
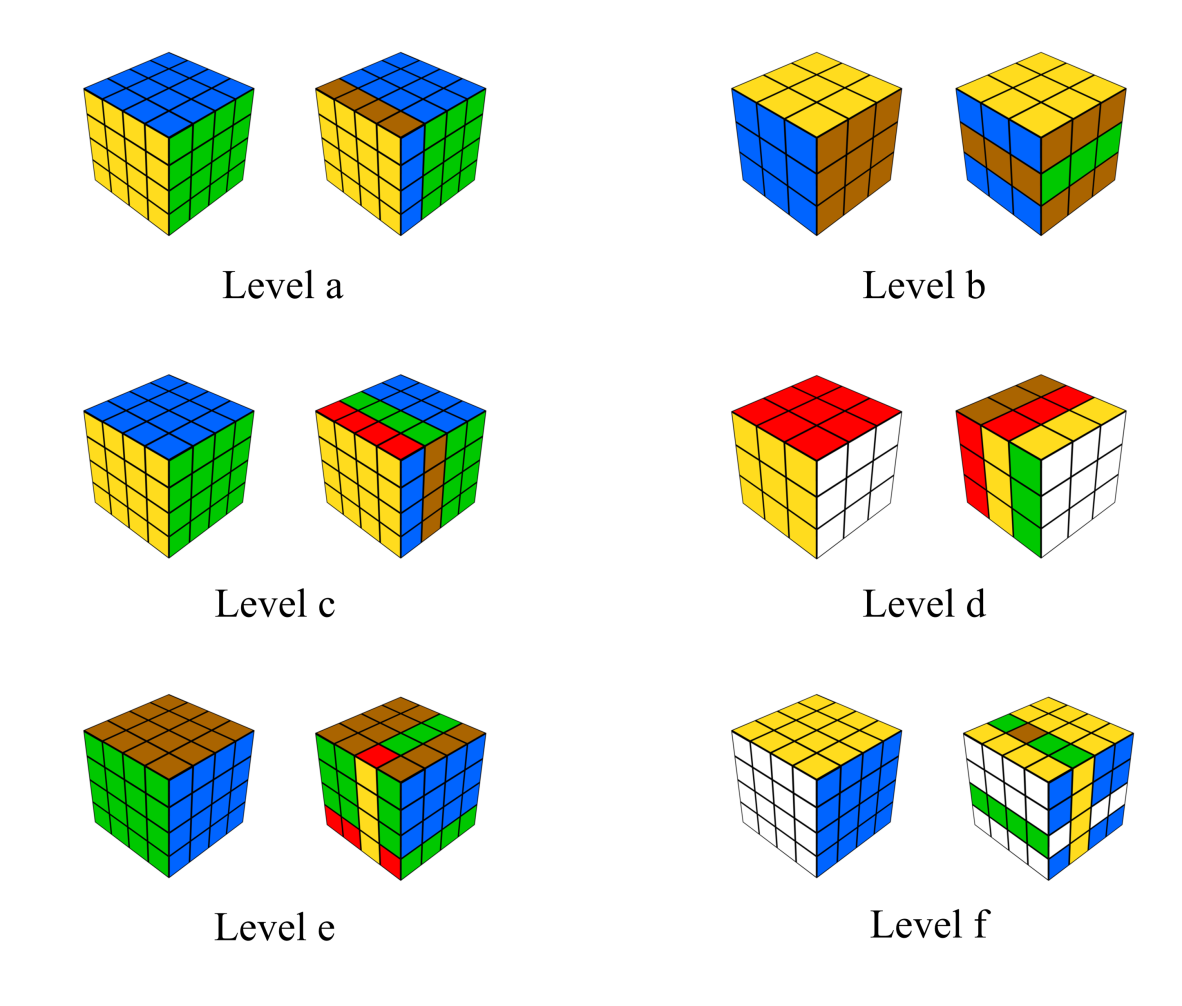
Possible sample items of the R-Cube-Vis Test for each difficulty level, ordered from easy (a) to difficult (f)

**Figure 2.**
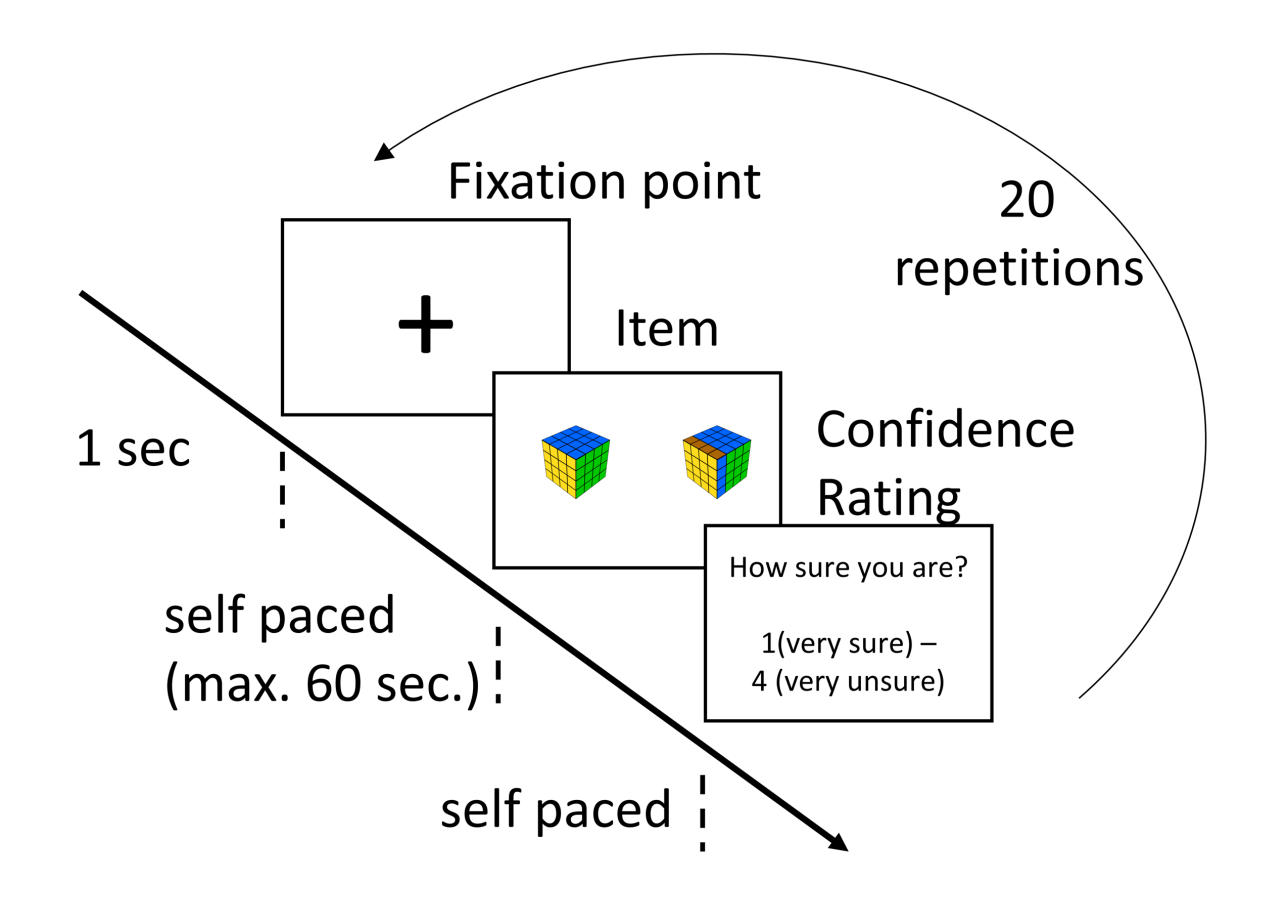
Description of one of the three blocks with 20 repetitions each (10 items for each of both levels, i.e., 5 possible and 5 impossible items). RT: reaction times.

The items can be assigned to six distinct difficulty levels that are conform to the linear logistic test model (Figure 1). The items were presented in three blocks. Within each block, the items belong to two neighbored difficulty levels (Level a and Level b; Level c and Level d; Level e and Level f) and were presented in random order. There were five possible and five impossible items per level. Before each block, a trial phase was conducted containing four cubes, one possible and one impossible of each difficulty level. The blocks were presented in the order of increasing difficulty. Each item was presented until an answer was given, maximum one minute. Before each item, a cross was presented for 1 second in the middle of the screen (Figure 2). The accuracy measure considers only all possible items and counts “1” for a correctly solved item and “0” for an incorrectly solved item.

Additional to the performance test, two simple fixation tasks, before the R-Cube-Vis Test and after the R-Cube-Vis Test, were conducted. Each fixation task presents nine black crosses, one after another, on an invisible grid with three rows and three columns in the middle of the screen. Each cross was presented for three seconds and was indicated by a square, which was presented immediately before the cross at the same position. The square rested also three seconds and changed its color from red, over yellow, to green each second.

Finally, the participants had to edit a questionnaire asking for sex, age, and major subject of studies.

### Procedure

After the instruction, the eye tracker was calibrated for each participant. Afterwards, the participants performed the first fixation task, then the R-Cube-Vis Test and then the second fixation task. At the end, they filled out the questionnaire.

### Apparatus

The used eye tracker was the Tobii TX300 (recording rate: 300Hz) embedded in an eye tracker unit with a screen (screen size: 23’’, aspect ratio: 16:9, resolution: 1920x1080 pixels). It was connected to the presentation software E-Prime 2.0 (30) with the “Extensions for Tobii” (29). The distance between eyes and eye tracker was M = 62.6 cm (SD = 5.71 cm) with the smallest distance of 51.1 cm and the largest of 73.2 cm.

### Data Preparation

The recommended scaled ICA values (8) were computed by the Workload RT software package (9). The IPA was computed according to the described algorithm (6) with an adaption of one of its parameters, which resulted in the closest similarity with the ICA (Factor = 0.8, Fehringer (10), using the same data set as in the present study). The IPA algorithm was programmed in python (39) using the packages pandas ( 25), numpy (38), and pywt (20). 

### Analyses

Instead of the classical null hypothesis significance testing, Bayesian statistic was applied to the data due to unknown effect sizes (see 34), especially for the fatigue effect and the difference values between both eyes. The effect sizes were estimated based on the means of the posterior distributions. All analyses were conducted with R statistics (31) using the package BayesFactor (27) for computing the Bayes factors.

First, the ordering of the accuracy values of the six difficulty levels of the R-Cube-Vis Test were compared with the expected accuracy ordering known from the validation studies (11). Second, the precision of both fixation tasks (before and after the R-Cube-Vis Test) were compared with each other to make sure that the participants performed both fixation tasks with the same task engagement as precondition for the following analysis of the fatigue effect. The precision was estimated as the standard deviation of the estimated gaze points during the fixation tasks and indicates how far a participant fluctuated around the fixated point.

The H_1_ hypotheses were formulated as directed hypotheses for the analyzed pair comparisons between different cognitive demanding tasks and as undirected hypothesis for the comparison between both fixation tasks testing the fatigue effects. All H_1_ hypotheses were formulated with the Cauchy distribution as default prior with the scale parameter r = 1 (33). The evidences were classified as anecdotal (> 1 and ≤ 3 for H_1_ or < 1 and ≥ 1/3 for H_0_), moderate (> 3 or < 1/3), strong (> 10 or < 1/10), very strong (> 30 or < 1/30), or extreme (> 100 or < 1/100), according to JASP Team (14) and Schönbrodt et al. (34). The Bayesian effect sizes were estimated as mean of the posterior distribution with a 95% confidence interval.

## Results

As expected, the accuracy decreased from the easiest level, Level a, to the most difficult level, Level f, whereby both easiest levels (Level a and b) showed comparable accuracy values (Table 1). The Bayes factors of the undirected t-tests to compare the precision between both fixation tasks (before and after the R-Cube-Vis Test) showed moderate evidence for equality in the x- and the y-coordinate, BF_10_ ≤ 0.16. Therefore, the task engagement in both fixation tasks can be assumed as equal.

**Table 1:** Mean (standard deviation) of the R-Cube-Vis Test accuracy measure differentiated for each difficulty level.

	**ACC**
**All levels**	.78 (.12)
**Level a**	.95 (.16)
**Level b**	.93 (.15)
**Level c**	.86 (.20)
**Level d**	.80 (.22)
**Level e**	.73 (.25)
**Level f**	.43 (.30)

**Figure 3.**
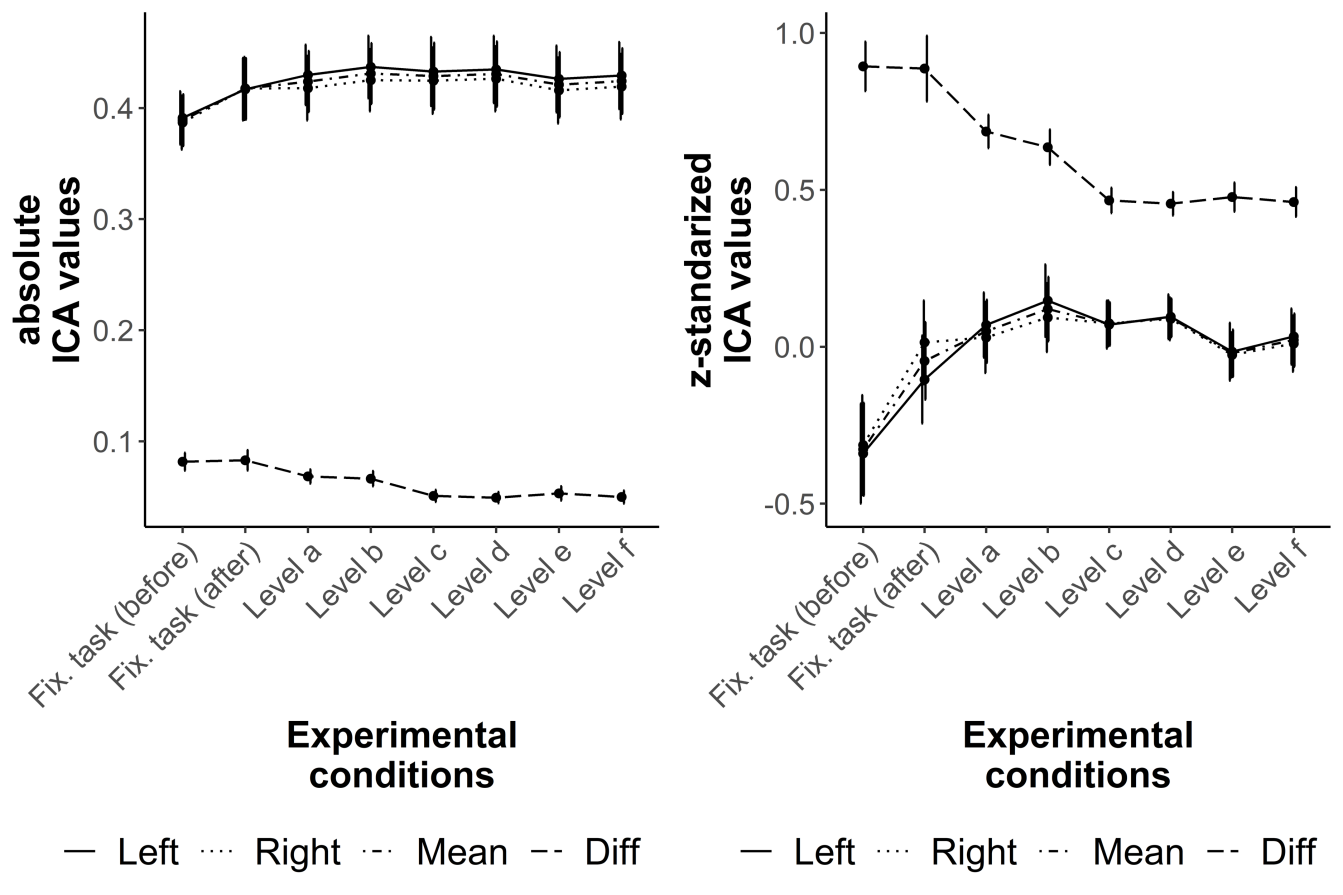
Distribution of the conventional (left) and the z-standardized (right) ICA values over both fixation tasks and the six difficulty levels of the R-Cube-Vis Test. The lines show the results for the left eye (Left), right eye (Right), their mean (Mean), and the difference between both eyes (Diff).

The distribution of the conventional ICA values over both fixations tasks as well as the six difficulty levels are comparable with the distribution of the z-standardized ICA values (Figure 3). However, the effects sizes of the considered pair comparisons of the Bayesian directed t-tests are either comparably the same or greater for the z-standardized than for the conventional values (Table 2). The distributions of the ICA values of the singles eyes (left, right) are almost the same as the distributions of the mean ICA values (Figure 3). Therefore, only the results of the mean and the difference ICA values (conventional and z-standardized) are considered in detail in the following.

**Table 2:** Effect sizes and Bayes factors for the considered pairwise comparison between of the conventional and z-standardized ICA values

		**Conventional**		**z-standardized**	
		**Mean**	**Difference**	**Mean**	**Difference**
**Fix. task 2**	*ES, Bayes*	.45 [.19; .71]	.03 [-.22; .28]	.42 [.16; .68]	-.01 [-.26; .24]
**- Fix. task 1**	*BF_10_*	17.36**	.11'	9.59*	.11'

**Level a**	*ES, Bayes*	.54 [.24; .80]	-.38 [-.62; -.09]	.55 [.25; .81]	-.63 [-.90; -.32]
**- Fix. task 1**	*BF_10_*	398.98****	8.52*	507.10****	2986.30****

**Level a**	*ES, Bayes*	.14 [.00; .31]	-.36 [-.61; -.08]	.16 [.00; .34]	-.46 [-.72; -.17]
**- Fix. task 2**	*BF_10_*	.20'	6.67*	.27'	43.75***

**Level b**	*ES, Bayes*	.21 [.00; .41]	-.15 [-.33; .00]	.20 [.00; .40]	-.23 [-.44; -.01]
**- Level a**	*BF_10_*	.60	.19'	.50	.66

**Level c**	*ES, Bayes*	.09 [.00; .23]	-.57 [-.82; -.26]	.08 [.00; .20]	-.75 [-1.02; -.42]
**- Level b**	*BF_10_*	.08''	568.64****	.07''	56687.94****

**Level d**	*ES, Bayes*	.13 [.00; .30]	-.16 [-.34; .00]	.13 [.00; .30]	-.13 [-.30; .00]
**- Level c**	*BF_10_*	.17'	.22'	.17'	.16'

**Level e**	*ES, Bayes*	.05 [.00; .13]	-.07 [-.17; .00]	.04 [.00; .12]	-.08 [-.20; .00]
**- Level d**	*BF_10_*	.03'''	.05''	.03'''	.06''

**Level f**	*ES, Bayes*	.17 [.00; .35]	-.21 [-.41; -.01]	.19 [.00; .38]	-.15 [-.33; .00]
**- Level e**	*BF_10_*	.30'	.49	.40	.20'

For each comparison, the effect size as mean of the posterior distribution of the Bayes statistics (ES, Bayes) and the Bayes factor (BF_10_) are reported. The evidence for H_1_ (difference) and H_0_ (equality) are marked as moderate (*/'), strong (**/''), very strong (***/'''), or extreme (****/''''). This marking refers only to the BF_10_ and not to the effect sizes.

The mean ICA values (conventional and z-standardized) are only able to differentiate between the fixation tasks (before, after) and the easiest level of the R-Cube-Vis Test (Table 2). There is a moderate to strong difference between the fixations task before and the fixation task after the R-Cube-Vis Test (BF_10_ ≥ 9.59), which might indicate a fatigue effect. Level a has only greater ICA values compared to the fixations task before the test with extreme evidence (BF_10_ ≥ 398.98). All other pair comparisons of the mean ICA values show only anectodical to very strong evidence for equality (BF_10_ ≤ .60). However, an unexpected decrease of the mean ICA values from Level d to Level e can be observed in Figure 3. The post-hoc conducted undirected Bayesian t-test resulted in a difference between the two levels with anecdotal evidence for the conventional and z-standardized values (BF_10_ ≥ 1.21) might indicating a decrease of the mean ICA values from the easier Level d to more difficult Level e.

The difference of the left and right eye’s ICA values shows the expected distribution with greater difference for less demanding tasks and lower differences for more demanding tasks (Figure 3). Similar as for the mean ICA value, the results of the z-standardized values have comparably the same or stronger evidence than of the conventional values ( Table 2). The difference of the z-standardized values of both fixation tasks (before, after) are greater than the difference value of Level a with very strong to extreme evidence (BF_10_ ≥ 43.75, Table 2). Remarkably, the presumable fatigue effect of the mean values indicated by larger values of the fixation task after the R-Cube-Vis Test compared to the fixation task before the test could not be found for the difference of the values of both eyes. In contrast to the mean values, the difference of the ICA values (conventional and z-standardized) are able to differentiate between the second of the easiest levels (Level b) and the first of the medium levels (Level c) with extreme evidence (BF_10_ ≥ 568.64, Table 2). This might be due to the different number of demanded transformation steps (1 vs. 2 rotated elements).

The post-hoc comparison between both levels with one transformation step (Level a and b) and the four levels with two transformation steps (Level c to f) showed an extreme evidence for inequality (BF_10_ ≥ 19852) for conventional and z-standardized difference values. All other pair comparisons of the difference values show anecdotical to strong evidence for equality (BF_10_ ≤ .66, Table 2). The pattern of results stayed the same if only correct items or only possible items were considered.

**Figure 4.**
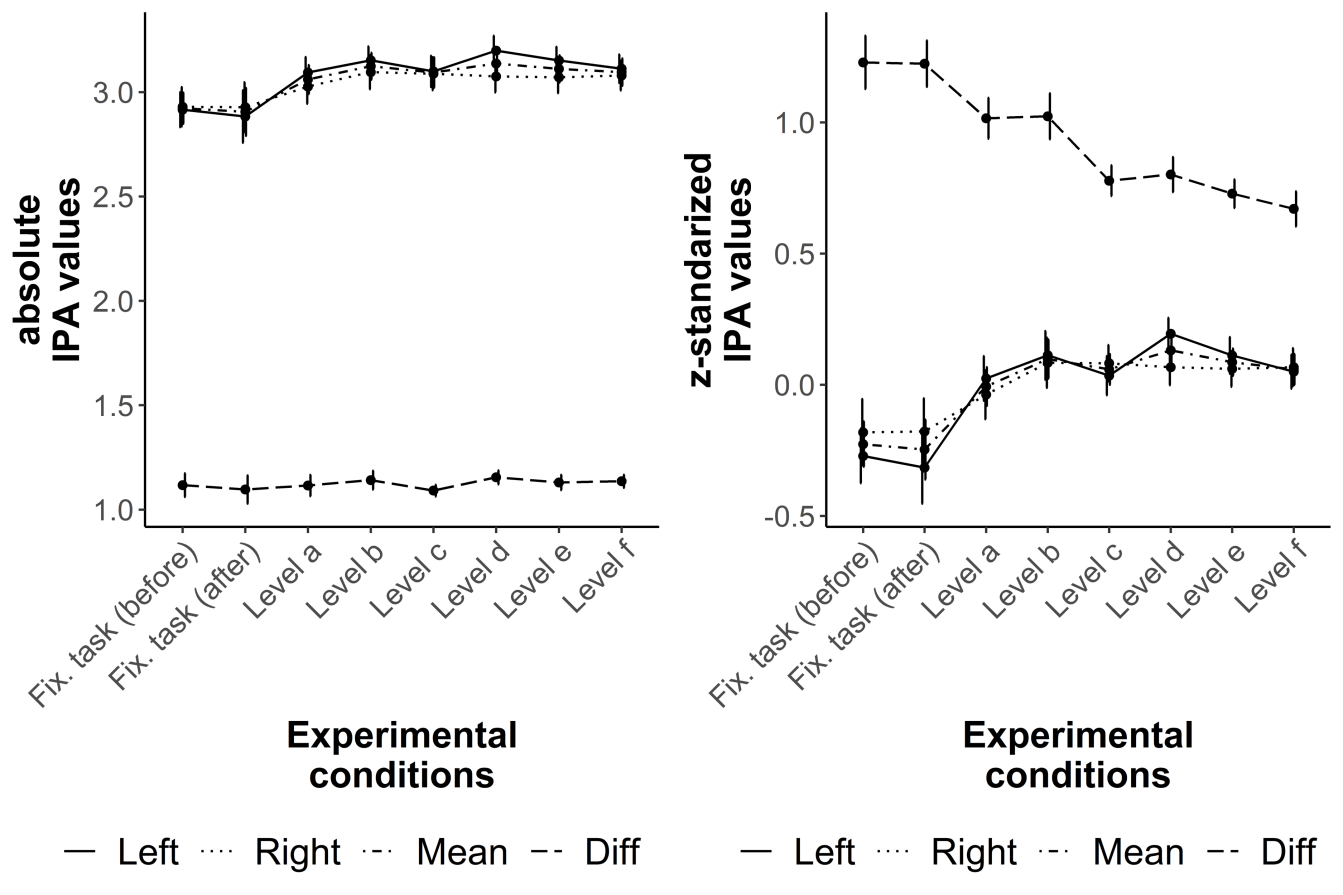
Distribution of the conventional (left) and the z-standardized (right) IPA values over both fixation tasks and the six difficulty levels of the R-Cube-Vis Test. The lines show the results for the left eye (Left), right eye (Right), their mean (Mean), and the difference between both eyes (Diff).

The results of the IPA values, conventional and z-standardized as well as mean and difference, are quite comparable to the results of the ICA (Figure 4 and Table 3). There is an increase of the mean values from the fixation tasks to Level a and comparable mean values between all difficulty levels of the R-Cube-Vis Test. However, the observed fatigue effect of the mean ICA values between the fixations task before and after the test could not be found for the mean IPA values. The z-standardized difference IPA values show a decrease from both fixation tasks to Level a and from Level b to Level c similar as it was found for the difference ICA values. The post-hoc comparison between Level a and b (one transformation step) against Level c to f (two transformation steps) showed extreme evidence for inequality (BF_10_ ≥ 175355884). All other pair comparisons have evidence for equality or only anectodical evidence for inequality (BF_10_ = 1.45 between Level d and Level e for the z-standardized values, Table 3). Similar as for the ICA values, only correct items or only possible items showed the same pattern of results.

**Table 3:** Effect sizes and Bayes factors for the considered pairwise comparison between of the conventional and z-standardized IPA values

		**Conventional**		**z-standardized**	
		**Mean**	**Difference**	**Mean**	**Difference**
**Fix. task 2**	*ES, Bayes*	-.05 [-.29; .21]	-.07 [-.32; .19]	-.03 [-.28; .22]	-.01 [-.26; .24]
**- Fix. task 1**	*BF_10_*	.11'	.12'	.11'	.11'

**Level a**	*ES, Bayes*	.47 [.17; .72]	-.11 [-.27; .00]	.43 [.17; .71]	-.43 [-.69; -.14]
**- Fix. task 1**	*BF_10_*	66.24***	.11'	32.85***	24.96**

**Level a**	*ES, Bayes*	.39 [.13; .67]	-.09 [-.22; .00]	.38 [.10; .64]	-.46 [-.73; -.18]
**- Fix. task 2**	*BF_10_*	13.96**	.08''	12.33**	49.87***

**Level b**	*ES, Bayes*	.29 [.04; .53]	.08 [-.20; .00]	.29 [.04; .54]	-.10 [-.25; .00]
**- Level a**	*BF_10_*	2.19	.06''	2.23	.10'

**Level c**	*ES, Bayes*	.07 [.00; .18]	-.27 [-.50; -.02]	.07 [.00; .19]	-.64 [-.90; -.33]
**- Level b**	*BF_10_*	.06''	1.29	.06''	3564.10****

**Level d**	*ES, Bayes*	.25 [.00; .46]	-.04 [-.12; .00]	.26 [.03; .50]	-.09 [-.22; .00]
**- Level c**	*BF_10_*	1.05	.03'''	1.36	.07''

**Level e**	*ES, Bayes*	.06 [.00; .18]	-.19 [-.39; .00]	.06 [.00; .17]	-.28 [-.50; -.03]
**- Level d**	*BF_10_*	.05''	.36	.05''	1.45

**Level f**	*ES, Bayes*	.08 [.00; .20]	-.10 [-.24; .00]	.07 [.00; .20]	-.23 [-.44; -.01]
**- Level e**	*BF_10_*	.06''	.09''	.06''	.68

*/' moderate, **/' 'strong, ***/''' very strong, ****/'''' extreme evidence for H_1_ (difference) respectively H_0_ (equality). This marking refers only to the BF_10_ and not to the effect sizes.

## Discussion

The goal of the present study was to investigate the Index of Cognitive Activity (ICA, 22) and the Index of Pupillary Activity (IPA, 6) as pupillary based indicators for cognitive workload systematically with respect to different cognitively demanding tasks, influence of fatigue, effect of standardization for controlling individual differences, and using difference values of the eyes instead of the mean. To this end, a performance test for visualization, the R-Cube-Vis Test (11), with six distinct difficulty levels was conducted to analyze the ICA/IPA behavior over different cognitive demanding tasks. The fatigue effect was investigated by two simple fixations tasks, one before and one after the R-Cube-Vis Test. Inter-individual differences in ability were controlled by z-standardization of the ICA/IPA values within each participant. Finally, additionally to the mean of the ICA/IPA values of both eyes, the changes of the variation of the pupillary response of both eyes were analyzed during these conditions by considering the absolute difference between the ICA/IPA values of both eyes. All analyzes were conducted using Bayesian statistics.

### Controlling for inter-individual differences

Previous studies showed that pupil diameter changes behave differently depending on the participants’ abilities and their expertise during a performance test (e.g. 12; 37; 40) and would, therefore, indicate capacity utilization rather than absolute processing demands (16). However, the maximum pupil diameter changes might also be different for all participants and, also, they might change with the amount of allocated resources. Therefore, the study also investigated z-standardized values to control for differences in the individual ranges. The results showed that this z-standardization is indeed able to control for these inter-individual differences. The z-standardized values (mean and difference of both eyes) of the ICA and IPA resulted in similar distributions as the conventional values but with comparable to greater effect sizes and stronger evidence in the Bayesian statistics. However, it might be important to note that the complete range of cognitive ability was addressed for presumably every participant in the present experimental setting. The fixation tasks demand nearly no (higher) cognitive activity, whereas the most difficult level of the R-Cube-Vis Test has an average accuracy on chance level and is, therefore, difficult for nearly each participant. Hence, it can be assumed that the cognitive capacity described by Just et al. (16) is completely exploited by each participant at some point during the experiment. Therefore, the z-standardization might be misleading, if only some participants would reach their maximal capacity.

### The mean ICA and IPA values as indicator for cognitive workload

The mean ICA and IPA values of both eyes (conventional and z-standardized) showed the expected effect between both fixation tasks and the easiest level of the R-Cube-Vis Test. In contrast to previous studies (e.g., 22; 35), differences between different difficulty levels could not be found for the conventional and z-standardized ICA and IPA values. One reason might be that accuracy per se is not a valid criterion for cognitive workload, but that even the easiest level with an average accuracy of 95% demands comparable cognitive resources (i.e., the mental manipulation of a cube) such as the more difficult levels. That would mean that the additional cognitive demand due to additional transformation steps of the more difficult levels (i.e., rotating two elements instead of one, crossed rotated elements) could not be captured by the mean ICA and IPA values. Another reason might be that the mean ICA and IPA values are only sensitive either for the increasing demand of other cognitive resources, such as the working memory (e.g., by measuring the digit span, 18) or for adding a complete new task (e.g., in a driving scenario with a secondary task, 35). 

### Indicating the fatigue effect

The general effect of fatigue is a decreasing of the pupil diameter and its changes (e.g. 1; 13). However, current studies showed constant or increasing pupil diameter changes if participants were engaged in solving a task (e.g. 28). In the present study, participants should stare at certain points in two fixation tasks before and after the conducted R-Cube-Vis Test. The comparison of the precision (separated for x- and y-coordinate) suggest that the participants had the same task engagement in both fixation tasks. The mean conventional and the z-standardized ICA values increased from the fixation task before the R-Cube-Vis Test to the fixation task after the R-Cube-Vis Test and, therefore, seems to be sensitive for fatigue. However, the ICA difference values as well as all IPA values (mean and difference values), stayed the same and indicated no fatigue effect. However, the underlying mechanisms for this difference cannot be determined based on the present study, since the algorithm of the ICA is not fully documented. Further studies focusing on fatigue effects utilizing further parameter changes of the IPA might provide more insights.

### Variability of the ICA and IPA values between the left and the right eye

Based on the general statement of Kahneman (17) and the results with heart rate variability (e.g. 4; 7; 24), it was assumed that the variability of the pupillary based indicators (ICA and IPA) between both eyes might be indicative for cognitive workload. The absolute difference values, calculated from the ICA and IPA values of the left and right eye supported this assumption. Generally, the more difficult the task is, the lower are the difference values. Similar as for the conventional values, the z-standardized values showed the greater effects with stronger evidence compared to the conventional values. The expected ordering with moderate to very strong evidence could be found between the fixation tasks and the easiest level of the R-Cube-Vis Test, such as with the mean values of both eyes. Additionally, the difference values changed also between Level b (one of the easiest levels) and Level c (one of the medium levels). Interestingly, there is a qualitative difference between both levels. In the easiest levels, the participants have to rotate only one element, whereas in the medium and difficult levels, the participants have to rotate two elements. This difference in the cognitive demand between these levels seems to be captured by the difference values of the ICA and IPA, but not if only the mean value of the eyes is considered.

Furthermore, in contrast to the mean ICA values, the difference values between both eyes are not sensitive for fatigue with moderate evidence for equality between the fixation tasks before and after the R-Cube-Vis Test.

### Limitations

Although the ICA and IPA are promising pupillary based indicators for cognitive workload, there are only few studies that have investigated these measures systematically. The reasons might be that the ICA is a patented and, therefore, not published algorithm and the IPA has been made publicly available only recently. Therefore, the found results in the present study have only weak support by previous studies and are partly explorative. This is particularly true for the fatigue effect.

### Conclusion

The present study investigated factors influencing the ICA and IPA values. The strength of the present study is its systematic analyzes that are necessary, if these (or similar) pupillary based measures should be utilized in application scenarios, where the specific meaning of these indicators is necessary to derive important decisions. Based on the found results, the following recommendation can be made for the usage of the ICA and IPA as indicators for cognitive workload. (1) Instead of the conventional ICA and IPA values, the z-standardized values, within each participant, should be considered for analyzes. (2) If it is the goal to detect fatigue effects, then the ICA values of the mean of both eyes should be used. (3) The absolute values of the difference of the ICA and IPA values between the right and the left eye should be used as fatigue independent measure to indicate cognitive workload. Moreover, the difference values are more sensitive to differentiate between different tasks with different cognitive demands.

However, due to the novelty of the results, further research is necessary to support the presented results and to test their generalization to other stimulus materials and for alternative pupillary based measures. Future studies should investigate whether the z-standardization also works if the limit of the cognitive resources is not reached and whether pupil dilations react differently to specific cognitive resources and to various demanding tasks. Also, it would be important to address the question how fatigue influences the investigated pupillary based indicators in detail.

If these indicators are better known with a deeper understanding of their behavior, the ICA and IPA seem to be very promising indicators that might be also used in more elaborative diagnostic applications as well as learning environments to gain more insights in the underlying cognitive processes.

## Ethics and Conflict of Interest

The author declares that the contents of the article are in agreement with the ethics described in http://biblio.unibe.ch/portale/elibrary/BOP/jemr/ethics.html and that there is no conflict of interest regarding the publication of this paper.

## Acknowledgements

The publication of this article was funded by the Ministry of Science, Research and the Arts Baden-Württemberg and the University of Mannheim. Furthermore, this work was supported by the University of Mannheim’s Graduate School of Economic and Social Sciences funded by the German Research Foundation. Also, I am very thankful for the many, very supportive discussions about the manuscript with Prof. Stefan Münzer who provided me a lot of valuable feedback to this work.
